# Dengue and Oropouche virus co-infection in a traveller from Cuba to Portugal

**DOI:** 10.1093/jtm/taaf051

**Published:** 2025-06-09

**Authors:** Líbia Zé-Zé, Joana Laranjinha, Vítor Borges, Ana L Graça, Daniel Sobral, João D Santos, Ana C Carvalho, Nuno R Faria, João P Gomes, Maria J Alves

**Affiliations:** INSA, Centre for Vectors and Infectious Diseases Research, National Institute of Health Doctor Ricardo Jorge, Avenida da Liberdade, 2965-575, Águas de Moura, Portugal; CECA, Center for the Study of Animal Science, ICETA, Institute for Agricultural and Agro-Alimentary Science and Technology, University of Porto, 4050-053, Porto, Portugal; Braga Local Health Unit, Braga Hospital, 4710-243, Braga, Portugal; INSA, Genomics and Bioinformatics Unit, Department of Infectious Diseases, National Institute of Health Doctor Ricardo Jorge, 1649-016, Lisbon, Portugal; Braga Local Health Unit, Braga Hospital, 4710-243, Braga, Portugal; INSA, Genomics and Bioinformatics Unit, Department of Infectious Diseases, National Institute of Health Doctor Ricardo Jorge, 1649-016, Lisbon, Portugal; INSA, Genomics and Bioinformatics Unit, Department of Infectious Diseases, National Institute of Health Doctor Ricardo Jorge, 1649-016, Lisbon, Portugal; Braga Local Health Unit, Braga Hospital, 4710-243, Braga, Portugal; MRC Centre for Global Infectious Disease Analysis, Department of Infectious Disease Epidemiology, School of Public Health, Faculty of Medicine, Imperial College London, Sir Michael Uren Hub, 86 Wood Lane, London W12 0BZ, United Kingdom; Departamento de Moléstias Infecciosas e Parasitárias e Instituto de Medicina Tropical da Faculdade de Medicina, Universidade de São Paulo, São Paulo, 05403-000, Brazil; INSA, Genomics and Bioinformatics Unit, Department of Infectious Diseases, National Institute of Health Doctor Ricardo Jorge, 1649-016, Lisbon, Portugal; Animal and Veterinary Research Center (CECAV), Faculty of Veterinary Medicine, Lusófona University - Lisbon University Centre, 1749-024, Lisbon, Portugal; INSA, Centre for Vectors and Infectious Diseases Research, National Institute of Health Doctor Ricardo Jorge, Avenida da Liberdade, 2965-575, Águas de Moura, Portugal; CECA, Center for the Study of Animal Science, ICETA, Institute for Agricultural and Agro-Alimentary Science and Technology, University of Porto, 4050-053, Porto, Portugal; ISAMB, Environmental Health Institute, Environment and Infectious Diseases Research Group, Av. Prof. Egas Moniz, Ed. Egas Moniz, Piso 0, Ala C, 1649-028 Lisboa, Portugal

**Keywords:** Co-infection, Oropouche, travellers, dengue

## Abstract

In 2024, unprecedented outbreaks of dengue and Oropouche were reported in the Americas. We describe a documented co-infection with dengue and Oropouche viruses in a 35-year-old traveller from Cuba detected in Portugal. Reverse transcription-polymerase chain reaction (RT-PCR) and next-generation sequencing confirmed both viruses. Our findings highlight the need for multiplex arboviral diagnostics in travellers from regions with concurrent outbreaks.

An estimated 5.7 billion people live in areas at risk for dengue (DENV), Zika (ZIKV) and chikungunya (CHIKV) viruses transmitted by *Aedes* spp. mosquitoes, posing a major global public health threat.^1^ Oropouche virus (OROV), an orthobunyavirus spread by *Culicoides* midges causes DENV/ZIKV/CHIKV-like illness.^2^ In 2024, unprecedented DENV and OROV outbreaks in Latin America and the Caribbean led to several imported cases in Europe.^3–5^

We report a 35-year-old Cuban man who presented to the emergency department in Braga, Portugal, on 2024 November 13, with a 24-h history of fever, myalgias, arthralgia, anorexia, mild retro-orbital pain and a dry cough. He arrived in Portugal from Cuba one day before symptom onset and had not travelled outside Cuba during the preceding 12 months. On observation, he was febrile (38.7°C), with a normal neurological examination and no other clinically significant findings. Blood tests showed leukopenia (total white cell count 2100/mm^3^; norm: 4300–10 800/mm^3^), lymphopenia (1100/mm^3^; norm: 1500–4500/mm^3^), thrombocytopenia (82 000/mm^3^; norm: 150 000–400 000/mm^3^), mildly elevated C-reactive protein (9.3 mg/L; norm: ≤5 mg/L) and serum transaminase levels: AST 75 U/L and ALT 30 U/L (norm: ≤35 U/L). The patient was discharged after 12 h of observation with symptomatic treatment. One month later, he was reassessed in an infectious diseases consultation and found to be in full clinical recovery.

**Figure 1:**
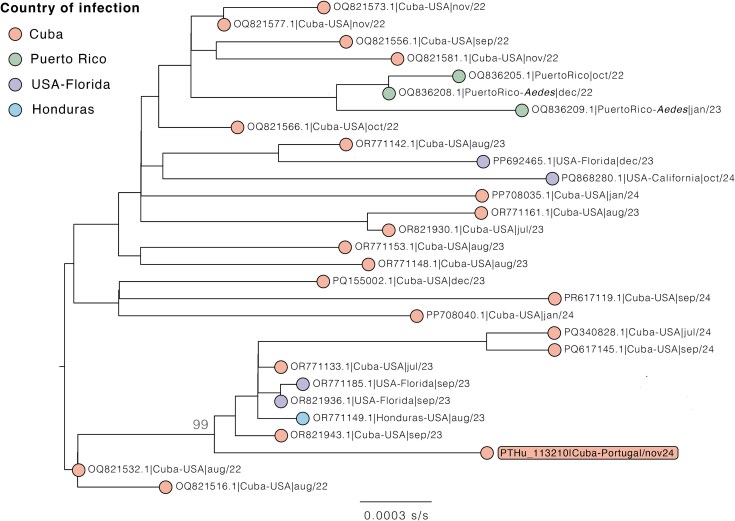
Maximum likelihood phylogenetic analysis of DENV3 detected in this clinical case. The figure provides a zoom-in view of the phylogenetic branch where the DENV3 detected in Portugal (highlighted in orange), following importation from Cuba, is positioned. Tips are coloured according to the country of infection (inferred from available travel history data) (Supplementary Table 1). This phylogenetic sub-branch (29 sequences) was first identified with Nextstrain phylogenetic analysis using 17 representative sequences from various DENV3 lineages/clades (n = 17), along 705 (near) complete sequences from clade “3III_B.3.2” available in the NCBI Virus database (https://www.ncbi.nlm.nih.gov/labs/virus/vssi/#/; TAXID 11069, as of 2025 April 2). The extended phylogenetic tree can be interactively explored on https://auspice.us/ using the JSON file and metadata provided as a Supplementary Dataset.

**Figure 2:**
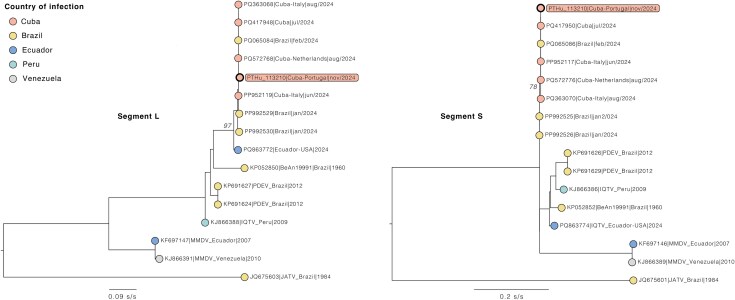
Maximum likelihood phylogenetic analyses of Oropouche virus L and S segments detected in this clinical case (highlighted), including sequences of strains reported in 2024 from Cuba, Brazil and Ecuador, and more divergent OROV strains, namely the historical prototype strain BeAn19991, Iquitos virus (IQTV), Madre de Dios virus (MMDV) and Perdões virus (PDEV). Jatobal virus (JATV) sequence was used as outgroup. Phylogenetic analyses were conducted as described in Supplementary Dataset.

Real-time RT-PCR confirmed DENV (Ct = 25.4; cut-off Ct≤40) and OROV (Ct = 37.1; cut-off Ct≤38)^6^ RNA in a blood sample; ZIKV and CHIKV were negative. Sanger sequencing using RT-PCR targeting partial capsid and pre-membrane region and NS5 pan-flavivirus amplification confirmed DENV3; OROV Sanger sequencing was inconclusive. Serology for DENV, ZIKV and CHIKV (IgM/IgG) was negative; OROV serology was unavailable. Illumina sequencing using the Viral Surveillance Panel v2 and MiSeq equipment generated ∼2 × 3.7 M reads. Bioinformatics analyses with INSaFLU-TELEVIR v.2.2.0 (https://insaflu.insa.pt/; default settings),^7^ produced a full DENV3 genome (147 596 mapped reads) and partial OROV L/S segments (12 mapped reads); these were deposited in ENA (Accession number: ERR14792439, BioProject: PRJEB87871). As this was the first time INSA handled this OROV lineage, cross-contamination can be excluded.

DENV3 (10 692 nt; NCBI accession number PV440223) belonged to lineage 3III_B.3.2,^8^ clustering closely with 2022–2024 strains from Cuba, Puerto Rico and the USA (Supplementary Dataset). A refined analysis of the 29 sequences within the identified phylogenetic sub-branch containing the patient’s DENV3 strain—incorporating country of infection inferred from available travel history data—is presented in Figure 1 (details in Supplementary Table 1). OROV partial sequences were assembled from segment S (321 nt, partial nucleocapsid protein) and segment L (419 nt in total, divided into two segments of 263 nt and 156 nt, of the RNA dependent RNA polymerase); these grouped with Cuban strains (Figure 2). Reoccurrence of symptoms, typical in 60% of patients infected with OROV,^9^ was not observed. The comparatively low OROV viral load suggests an earlier OROV infection, with acute DENV3 infection occurring thereafter (Supplementary Material). Negative DENV/ZIKV serology support a primary DENV infection. Genomic sequencing generated a full DENV3 and partial OROV L and S segments, all closely related to Cuban strains; the later were outside the RT-PCR S-segment amplicon, independently confirming co-infection. Given OROV’s tri-segmented genome and the circulation of reassortant strains, routine molecular assays can miss emerging variants. Our findings underscore the importance of equipping national reference laboratories beyond endemic regions with multiplex arboviral diagnostics and next-generation sequencing to screen travellers and migrants from outbreak areas, particularly as the geographic range of arboviruses continues to expand.^4^^,^^5^

## Supplementary Material

Supplementary_Table_1_taaf051

Ze_Ze_etal_Supplementary_Materials_Information_taaf051

SupplementaryFile_1_3_DENV3_Portugal_2024_genome_taaf051

SupplementaryFile_2_3_DENV3_Portugal_2024_genome_metadata_taaf051

SupplementaryFile_3_3_DENV3_Portugal_2024_genome_tip-frequencies_taaf051

## Data Availability

The data underlying this article are available in the European Nucleotide Archive (ENA) [BioProject accession no. PRJEB87871], and can be accessed with [ERR14792439], and in GenBank Nucleotide Database at [https://www.ncbi.nlm.nih.gov/nucleotide/], and can be accessed with [PV440223 (DENV3, complete genome) and PV446629 (OROV, nucleocapsid protein, partial sequence)], and in its online Supplementary Material.
