# Supplementary material for: Dengue and Oropouche virus co-infection in a traveller from Cuba to Portugal
Source: J Travel Med. 2025 Jun 9;32(7):taaf051. doi: 10.1093/jtm/taaf051 (PMC12640871; doi:10.1093/jtm/taaf051)
Supplement: Ze_Ze_etal_Supplementary_Materials_Information_taaf051 [file ze_ze_etal_supplementary_materials_information_taaf051.pdf]

Supplementary Materials

Supplementary Dataset

The Supplementary dataset files includes the results of the phylogenetic and geotemporal analysis conducted using DENV3 Nextstrain workflow available at INSaFLU-TELEVIR platform (https://insaflu.insa.pt/; https://github.com/INSaFLU/dengue, as of 2025 04-02)[1].

To phylogenetically place the DENV-3 sequence detected in Portugal (following importation from Cuba) within the current global genetic diversity of DENV-3, the analysis included 17 representative sequences from various DENV3 lineages/clades (n=17), along 705 (near-)complete sequences from clade "3III_B.3.2" available in the NCBI Virus database (https://www.ncbi.nlm.nih.gov/labs/virus/vssi/#/; TAXID 11069, as of 2025-04 02). DENV lineage classification was performed with Nextclade v 3.12.0 (https://clades.nextstrain.org/; consulted on 2025-04-02).

The tree can be interactively explored on https://auspice.us/ (first drag&drop both JSON files, and then the metadata TSV file).

NOTE: *In this global Nextstrain analysis[1], geolocation refers to the country of detection, not necessarily the country of infection, as travel history data was not available for all sequences in the dataset. However, a refined phylogenetic analysis of the sub-branch containing the DENV-3 sequence reported in this study is presented in **Figure 1**, where the country of infection is indicated (as detailed in **Supplementary Table 1**). Article readers can further explore and color the Nextstrain tree using the "origin_infection" and "travel_history" fields indicated in the metadata table for the 29 sequences included in **Figure 1**. Phylogenetic analyses presented in **Figure 1 and 2** were conducted using IQTREE2[2] using ModelFinder Plus[3] for optimal model selection (-m MFP) and 1000 ultrafast bootstrap[4] replicates to assess branch support (-bb 1000).*

Data analysis and discussion regarding co-infection timeline

Under control laboratory procedures (e.g. to avoid sample/RNA degradation; rigorous protocols; calibrated micropipettes, etc) Ct values should be a direct measurement of viral load, and the values that we have obtained one day after symptoms onset are significantly higher for DENV and close to positivity threshold for OROV. Considering that for DENV viraemia (and RNA detection) peaks close to symptoms onset[6], and at this point specific DENV IgG should be detected in secondary infections, our results (IgM and IgG negative for DENV) suggests that the onset of symptoms data should be mostly related to DENV infection. The patient reported no recent vaccinations, including for dengue. Vaccination status for yellow fever and measles was unknown.

For OROV, RT-PCR detection in blood has been reported several weeks after symptoms onset, with relapsing symptoms reported in almost 60% of OROV patients, between one to three weeks after symptoms onset, and lasting two to three weeks (mostly fever, myalgia, asthenia, dizziness and retro-orbital pain)[7,8]. Several studies report positive RT-PCR long after symptom onset, unrelated to symptom relapses (40-95 days after symptoms onset) [7,8].

Patient blood and urine samples collected 45 days after symptoms onset were tested by RT-PCR and were negative for DENV and OROV.

Data reported for patient DENV diagnosis (samples collected one day after symptoms onset: whole blood sample positive for DENV RNA [Ct 25.4] and serum negative for DENV, IgM and IgG), data reported for OROV (whole blood collected one day after symptoms onset positive for OROV RNA [Ct 37.1], and whole blood and urine samples collected 45 days after symptoms onset negative for OROV RNA), and according to data reported by Colavita et al. (2025)[7], in Supplementary data (available at: https://www.thelancet.com/cms/10.1016/S1473-3099(24)00798-9/attachment/66c60097-b66d-4823-a91f-019f93a3832f/mmc1.pdf), this co-infection case data suggests that the patient may have been primarily infected with OROV probably at least two weeks before DENV3 infection.

References

1. Santos JD, Sobral D, Pinheiro M, et al. INSaFLU-TELEVIR: an open web-based bioinformatics suite for viral metagenomic detection and routine genomic surveillance. Genome Med 2024;16:61.

2. Hadfield J, Megill C, Bell SM, et al. Nextstrain: real-time tracking of pathogen evolution. Bioinformatics 2018;34:4121-4123.

3. Minh BQ, Schmidt HA, Chernomor O, et al. IQ-TREE 2: New Models and Efficient Methods for Phylogenetic Inference in the Genomic Era. Mol Biol Evol 2020;37:1530-1534.

4. Kalyaanamoorthy S, Minh BQ, Wong TKF, et al. ModelFinder: fast model selection for accurate phylogenetic estimates. Nat Methods 2017;14:587-589.

5. Minh BQ, Nguyen MA, von Haeseler A. Ultrafast approximation for phylogenetic bootstrap. Mol Biol Evol. 2013;30:1188-95.

6. Dengue: Guidelines for Diagnosis, Treatment, Prevention and Control: New Edition. Geneva: World Health Organization; 2009. 4, LABORATORY DIAGNOSIS AND DIAGNOSTIC TESTS. Available from: https://www.ncbi.nlm.nih.gov/books/NBK143156/

7. Colavita F, Carletti F, D'Abramo A, et al. Prolonged detection of Oropouche virus RNA in whole blood samples [published correction appears in Lancet Infect Dis. 2025;25:e137]. *Lancet Infect Dis*. 2025;25:e11-e12.

8. Gourjault C, Pezzi L, Doudier B, et al. Persistence of Oropouche virus in body fluids among imported cases in France, 2024. *Lancet Infect Dis*. 2025;25:e64-e65.
